# Toadyism

**Published:** 1856-04

**Authors:** 


### TOADYISM.

We are grieved to see our Medical Journals giving importance to the accouchment of the Empress of the French, and the birth of a son and heir to Royalty. A nation, and in a particular manner, the medical *profession* of that nation, who sneer at the efforts of the medical men of this country, do not deserve such consequence at our hands.

But was there anything peculiar in this case? Was not the impregnation brought about by the ordinary process? Did not the development go on in the usual way? Was not there the ordinary abdominal tumor? Did not expulsion take place by the force of the unterine fibers? Was there anything peculiar in the position or the presentation? Or was the child unlike any *other child?* It has not been duly chronicled if there was. Now we undertake to say that there was, in this country, on the very day on which that young *Gaul* was born, a hundred better specimens of human nature than this gilded, petted, courted, and worshiped sprig of royalty, and we undertake also to say that in this city, "away in the west" there are dozens of finer sovereigns than can be found in *any* royal cradle, and yet, when born, no public display of *foolery* followed, and royalty will never be the wiser until they quarrel with us, when their wisdom may be dearly bought.

